# TiArA: A Virtual Appliance for the Analysis of Tiling Array Data

**DOI:** 10.1371/journal.pone.0009993

**Published:** 2010-04-01

**Authors:** Jason A. Greenbaum, Erika Assarsson, Jo L. Chung, Steven Head, Alessandro Sette, Bjoern Peters

**Affiliations:** 1 Division of Vaccine Discovery, La Jolla Institute for Allergy and Immunology, La Jolla, California, United States of America; 2 DNA Array Core Facility, The Scripps Research Institute, La Jolla, California, United States of America; King Abdullah University of Science and Technology, Saudi Arabia

## Abstract

**Background:**

Genomic tiling arrays have been described in the scientific literature since 2003, yet there is a shortage of user-friendly applications available for their analysis.

**Methodology/Principal Findings:**

Tiling Array Analyzer (TiArA) is a software program that provides a user-friendly graphical interface for the background subtraction, normalization, and summarization of data acquired through the Affymetrix tiling array platform. The background signal is empirically measured using a group of nonspecific probes with varying levels of GC content and normalization is performed to enforce a common dynamic range.

**Conclusions/Significance:**

TiArA is implemented as a standalone program for Linux systems and is available as a cross-platform virtual machine that will run under most modern operating systems using virtualization software such as Sun VirtualBox or VMware. The software is available as a Debian package or a virtual appliance at http://purl.org/NET/tiara.

## Introduction

Tiled microarrays have become increasingly ubiquitous in recent years, used in experiments ranging from Chromatin Immunoprecipitation on a chip (ChIP-chip) analysis to transcript identification. In contrast to traditional microarrays, which contain a relatively low number of probes, tiling arrays include probes that are spaced at regular intervals along a DNA sequence, often overlapping, providing the user with a continuous hybridization signal along the entire length of a DNA molecule (often an entire genome).

To date, only a handful of user-friendly software tools have been published for the analysis of tiled microarray data [1], [2], [3]. These tools focus on the identification of transcriptionally active regions, while TiArA's main focus is the summarization of transcription data based upon previously existing annotations. Additionally, the array platforms supported by the currently available methods are limited to the publically available chips, while many tiling arrays have custom, non-standard designs. TiArA aims to be an integrative platform upon which the user can perform the background subtraction, data normalization, and generate a summary report all in one application for any Affymetrix chip including customized designs.

TiArA was developed as a disparate collection of Perl and R [4] scripts written on Ubuntu Linux. To allow end users that are not comfortable with command-line script execution, the scripts were tied together with a GIMP Toolkit (GTK) graphical user interface (GUI). In planning its distribution, we have exploited the ability of the modern desktop computer to run multiple operating systems in parallel (i.e. virtual machines). This permits us to distribute the program together with all libraries and applications, including a MySQL backend, required for its execution in a single file to users of Windows and MacOS. This packaging of machine-specific code into a virtual appliance (a virtual machine developed for a highly specific use), provides a completely new avenue of software distribution that is particularly useful in the field of bioinformatics.

We present here TiArA, a user-friendly software platform for tiling array analysis. We describe the empirical background estimation and subtraction processes, data normalization, data summarization, and a general method for distribution of complex bioinformatics software packages.

## Results and Discussion

### Scope and functionality

TiArA was originally developed for the analysis of vaccinia virus whole-genome tiling array data [5]. This was the first study of its kind, analyzing the gene expression of a complex virus upon infection of a host cell. TiArA provides the user with an intuitive interface for preprocessing and summarization of Affymetrix tiling array data. Moreover, any Affymetrix tiling array is amenable to analysis using TiArA, including customized arrays using their array layout as described in the Affymetrix TPMAP file format.

Specifically, TiArA performs a background subtraction based upon an empirical measurement of the background signal, normalizes the dynamic range of signals, performs quantile normalization over multiple arrays, and produces a summary of expression levels and associated P-values based upon a given genome annotation file. Additionally, the user is able to export the probe signal intensity data in WIG file format for viewing in genome browser applications. The user also has the option to save the data for further processing and/or exploration in R. A detailed description of its use, including screenshots, can be found in the help manual which is included in the Supporting Information (Text S1).

### Background subtraction and data normalization

**Figure 1:**
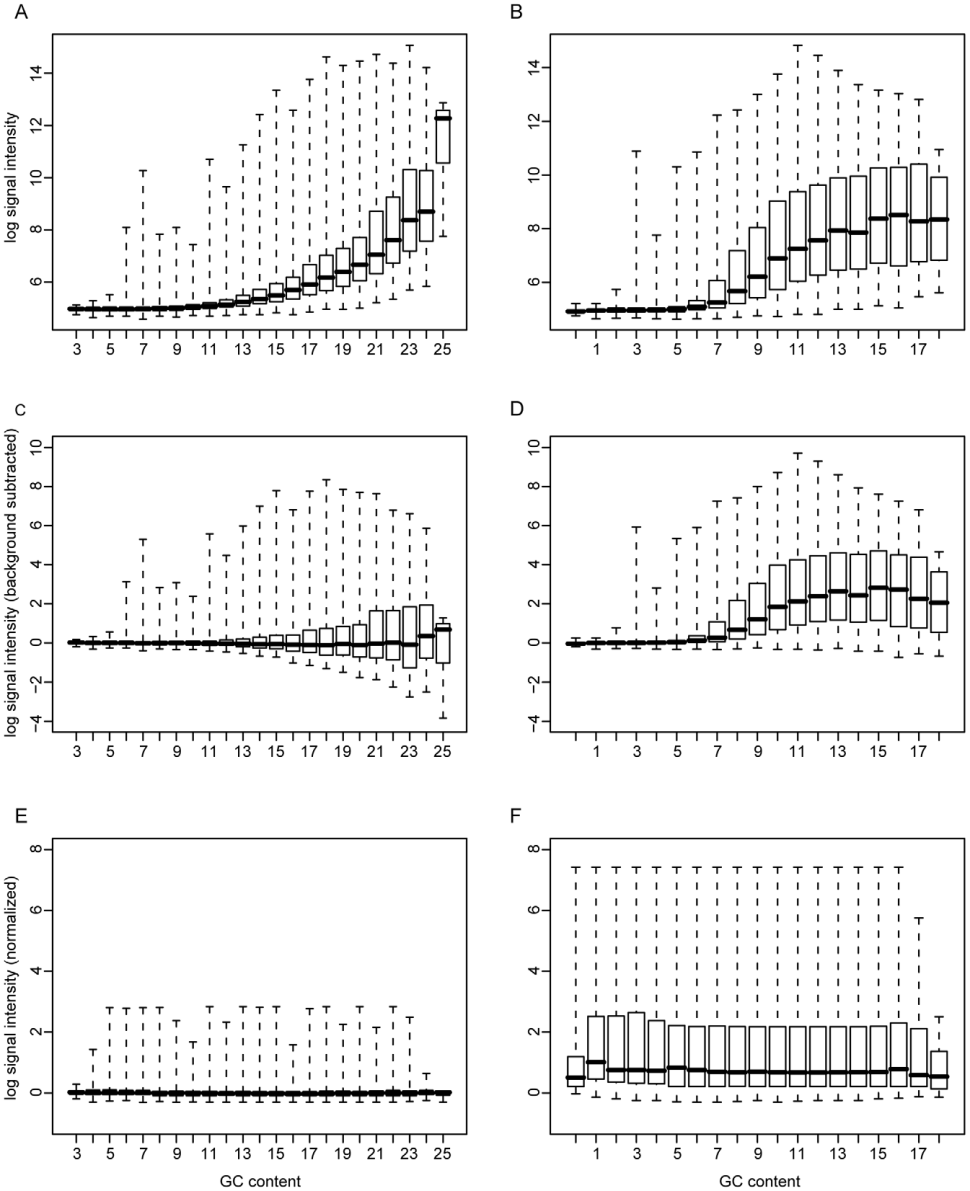
Correlation of signal intensity with GC content. RNA from vaccinia-infected cells was hybridized to the chip. Each boxplot shows the signal intensity of probes on the chip grouped by their GC content. The boxes are drawn extending from the 25^th^ to the 75^th^ percentile with the medians marked by horizontal lines. The whiskers extend to the minima and maxima of the distributions. A. Nonspecific background probes (12,192) prior to subtraction. B. Vaccinia-specific probes (97,344) prior to subtraction. C. Nonspecific background probes after subtraction of median GC signal. D. Vaccinia-specific probes after subtraction of median background signal. E. Nonspecific background probes after background subtraction and distribution mapping to a GC content level of 8. F. Vaccinia-specific probes after background subtraction and distribution mapping to a GC content level of 8.

The unique aspect of our technique involves the selection of probes used for background subtraction and normalization. In a typical Affymetrix microarray analysis, the background signal is estimated using a set of mismatch probes [6]. That is, every probe on the array has a corresponding probe that is identical except for its central nucleotide. This paradigm has several drawbacks. Most apparent is that this instantly doubles the size requirement of the chip, limiting the number of interesting probes and adding significant monetary cost. In addition, we found that the probe expression levels, typically expressed as fold difference between probe and mismatch probe are problematic to compare between ORFs with differing GC content. Our approach specifically addresses these deficiencies while maintaining a robust methodology. It is based upon the observed correlation between probe signal intensity and its GC content. Specifically, our approach requires a set of nonspecific randomized sequences with varying GC content (3–25 per 25-mer probe) to be synthesized on the array. In the analysis, these probes are grouped by their GC content and their median signal intensity is calculated. A similar methodology is described on the Affymetrix website related to exon array background correction [7], but is not available in the Tiling Analysis Software package [1]. As can be seen in Figure 1A, probes with higher GC content have both a higher signal, and a larger dynamic range.

**Figure 2:**
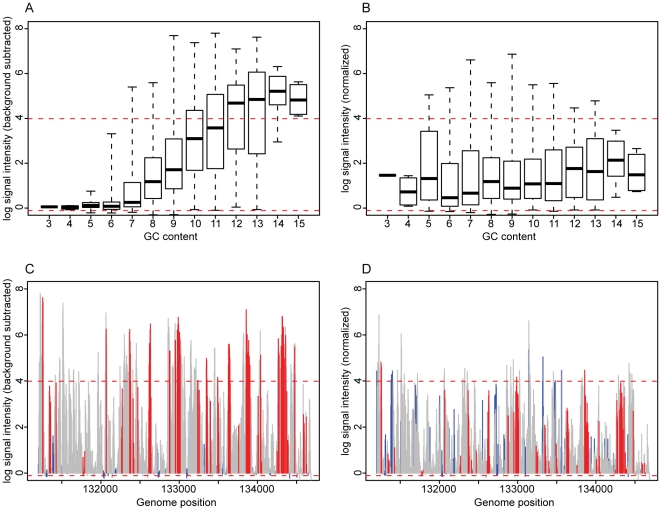
Removal of GC-dependant signal intensity. A. Probes that hybridize to a transcript of VACWR144 (N = 867) are grouped by their GC content and presented as a boxplot as in Figure 1. The dashed, red lines indicate 10^th^ and 90^th^ percentiles of probe signal intensity for a GC content of 8. All probes are normalized to this probe distribution in B, forcing the distributions into a common range and eliminating the signal GC dependence. C. The background-subtracted signal intensities of all probes that hybridize to VACWR144 are plotted along their genomic coordinates. Probes are colored according to their GC content (high = red, low = blue, medium = grey). D. The normalized signal intensities of the probes plotted in C, with the same color scheme.

We correct for this bias in a two-step procedure. First, the background signal for probes of a given GC content is estimated to be the median value of the nonspecific probes of the same GC content. Figures 1C and 1D show the background subtracted nonspecific and vaccinia-specific probes. Second, to adjust for differences in the dynamic ranges of the signals, the distributions were mapped to the distribution of probes with the best representation (i.e. the set of probes with the highest population). For the vaccinia array, there were 16,007 probes with a GC content of 8, more than for any other probe set. The mapping was accomplished using R's built in “quantile” and “ecdf” functions which essentially rank the value of each point within each distribution, and assign them their correspondingly ranked value in another distribution. Figure 2 illustrates the effect of this normalization process. More details on its implementation can be found in the Materials & Methods section.

In Fig. 2, the distributions of signal intensities for all probes within ORF VACWR144 are plotted after background subtraction (2A & C) and subsequent normalization (2B & D). After background subtraction, a dependence on the GC content of the probe signal is still clearly observed, with probes of a higher GC content giving a stronger signal. As all of the probes in this plot are specific for the same transcript, we assume that this variance cannot be due to true differences in transcript abundance, but to an artifact of sequence differences. In Fig. 2C & D, the probes with high GC content are colored in red, low GC content are blue, and average GC content are grey. Note that before normalization (2C) the majority of probes with a high GC content have a very high signal intensity, while those with a low GC content have a correspondingly low signal intensity. After normalization (2D), the intensities of probes at both extremes are brought into a common range, allowing for direct comparison among the signal intensities of probes of divergent composition.

The methodology described here has several advantages over the canonical mismatch probe subtraction. Perhaps its greatest is the need for a much smaller set of probes for subtraction, saving precious space on the array for more interesting probes. For example, on our vaccinia tiling array, there are 97,344 vaccinia-specific probes and the same number of mismatch probes. However, with a set of only 12,192 randomized sequence probes, we are able to perform a robust background subtraction and normalization. This potentially saves 85,152 spots on the array for additional probes and affords the possibility of cutting costs by moving to a smaller platform altogether. As the number of mismatch probes required for background subtraction scales linearly with the number of probes of interest on the array, the amount of space saved by this method becomes more pronounced as the number of features on the chip increases. Further, it is possible that a smaller set of background probes is sufficient for performing a robust normalization, but this remains to be tested.

Additionally, the randomized sequence probes have little sequence similarity to the probes of interest, making the calculation truly a measure of nonspecific background and minimizing the risk of over-estimation.

### Data summarization and significance testing

TiArA gives the user the option to summarize the probe-level data at the genomic annotation level (e.g. ORFs, CDS, genes, etc.). This is accomplished by pooling all probes that lie completely within each annotation, as denoted in a given Genbank file. The mean and median probe signals within each annotation are calculated and the significance of expression is assessed using the binomial distribution, as in [8]. The data are then summarized in tabular format and are exported to an Excel spreadsheet.

### Comparison with PM-MM background subtraction

**Figure 3:**
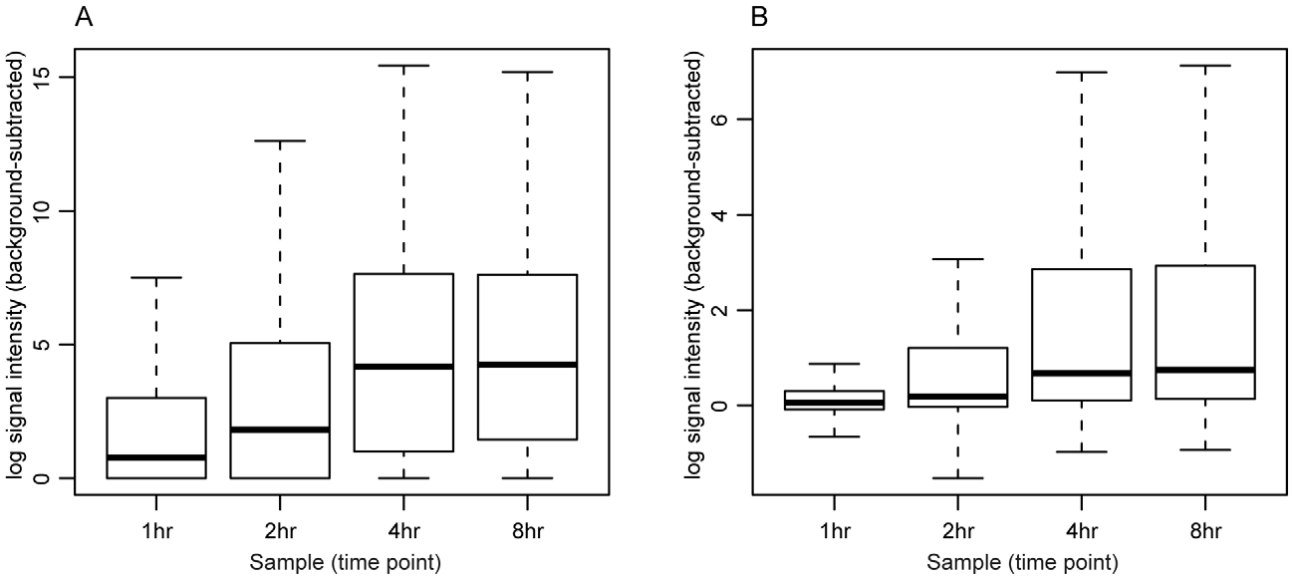
Distribution of TAS- and TiArA-processed signal intensities. A. The distributions of probe signal intensities are shown for each sample of TAS-processed (A) and TiArA-processed (B) data.

An analysis comparing the probe intensity levels and expression p-values obtained through this method and the canonical perfect match minus mismatch (PM-MM) method was performed. Table 1 summarizes the results of the probe intensity correlation between the two methods. Overall, the methods are very highly correlated, with an average Pearson correlation coefficient among the 4 samples of 0.95. Figure 3 illustrates the probe intensity distributions for the TAS-normalized and TiArA-normalized samples. Although the absolute values obtained with either method differ, the relative intensities among the samples are similar. Further, if we extrapolate to the ORF-level as opposed to the probe level, the binary classification of “expressed” or “not expressed” are nearly identical (Table 2, Dataset S1).

**Table 1:** Pearson correlation between TAS-processed and TiArA-processed probe signal intensity data.

Sample	Correlation
1 hr	0.90
2 hr	0.96
4 hr	0.96
8 hr	0.96
**Average**	**0.95**

**Table 2:** Number of ORFs detected as significantly expressed with TAS- and TiArA-processed data.

	TAS	TiArA
Sample	ORFs expressed (P<1e-4)	ORFs expressed (P<1e-4)
1 hr	73	76
2 hr	138	137
3 hr	139	138
4 hr	132	131

### Virtual machine packaging and distribution

As many bioinformaticians tend to code in different languages, using varying libraries, the code can become highly fragmented and machine-specific. Distributing these programs among several different machines could require hours of manual setup and troubleshooting. As an example, TiArA depends directly upon 18 other software packages. TiArA is available as a .deb package that can be installed on Ubuntu 8.04 or later. However, we have developed a virtual machine for its distribution which circumvents many of the installation issues and enables a great deal of customization. Alternatively, distribution as a Bioconductor [9] package was considered. However, the tight integration between R, Perl, and the MySQL database backend is more readily achieved through the development of a customized virtual machine.

A virtual machine (VM) can be configured precisely as needed by the developer and distributed to users of the application. This allows the application to be executed on any computer capable of running virtualization software, such as the open source Sun VirtualBox or the proprietary VMware Player/Server/Fusion. This includes all major platforms: Windows, MacOS, *NIX. The TiArA VM is distributed in Open Virtualization Format (OVF) and can be imported into Virtualbox or VMware.

The TiArA virtual machine runs the Ubuntu 8.04 operating system. The application itself is packaged as a .deb file, allowing us to take advantage of Ubuntu's native package manager and to automatically push updates to users of the software. The desktop is also highly customized with links to help files and the ability to email the developers.

The main downside in distributing a virtual appliance is the increased download size, which is 1.6 GB for TiArA. However, as broadband internet connections have become ubiquitous, this is no longer prohibitive. Moreover, this should cause far less traffic as compared to hosting TiArA as a server where users would regularly upload and download large data files.

### Summary

We have developed a program for the analysis of tiling array data, TiArA, which is packaged as a virtual appliance and uses an empirical estimation of background based upon probe GC content. The background subtraction methodology nearly doubles the space available on the array for probes of interest while producing intensity values that are consistent with those of the PM-MM method. The virtual appliance distribution schema ensures that the software is configured correctly and allows the program to run on almost any modern computer system.

## Materials and Methods

### Background subtraction and data normalization

All expression data were first log_2_ transformed. A total of 12,192 nonspecific probes were grouped according to their GC content, ranging from 3 to 25. The median signal intensity from each group was calculated and subtracted from all of the probes on the array with the same GC content. The process can be summarized as follows:
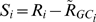



where *S_i_* is the background-subtracted signal intensity of probe *i*, *R_i_* is the raw signal intensity of probe *i*, and 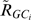 is the median raw signal intensity for all nonspecific probes with the same GC content as probe *i*.

After this nonspecific background subtraction, a specific component of GC-dependant signal was observed (Fig. 2A). This effect was minimized by normalizing the probe signals onto the probe signal distribution of the most well-represented GC content. For example, on the vaccinia array there are 16,007 vaccinia-specific probes with a GC content of 8, which is more than for any other level of GC content. The following R pseudo-code illustrates how this process is performed for mapping the signal intensity of probes with a GC content 4 to the signal intensity of probes with a GC content of 8:

subtracted_ecdf[gc_content  =  =  4] <-

ecdf(subtracted_signal[gc_content  =  =  4]);

normalized_signal[gc_content  =  =  4] <-

quantile(subtracted_signal[gc_content  =  =  8],

subtracted_ecdf(subtracted_signal[gc_content  =  =  4]));

The resulting probe signals are quantile normalized against the other arrays in the data set as in [10].

### R/Perl communication

TiArA uses a Perl subroutine to read in the user's data files and perform the background subtraction. Data are passed to an R socket server that is launched at program startup.

### Virtual machine configuration and customization

The virtual machine was created as described in the Sun Virtualbox User Manual [11]. Ubuntu 8.04 was installed as the operating system and a Debian package file (.deb) was created (tiara-desktop.deb) to act as a meta-package. Updates to the meta-package could include additional dependencies which would automatically be installed on the users' machine. A custom Debian repository, maintained at La Jolla Institute for Allergy and Immunology, is listed in the/etc/apt/sources.list file, allowing seamless upgrades in the background. The meta-package file also includes a set of maintenance scripts allowing for reconfiguring the virtual machine to its original state, connecting the host system disk to the virtual machine, and creating user profiles.

### Source code

All Perl and R source code is available on the virtual machine or after installation of the Debian package in the path ‘/opt/tiara’.

## Supporting Information

Text S1 TiArA User Guide. This is a comprehensive manual detailing the installation and use of TiArA.
(0.64 MB PDF)

Dataset S1 ORF expression comparison for TAS- and TiArA-processed data. Each sample was processed with both TiArA and TAS. The resulting median and mean intensities per ORF are listed here. Additionally, a P value for expression above background is reported for each ORF.
(0.18 MB XLS)
