# Supplementary material for: TiArA: A Virtual Appliance for the Analysis of Tiling Array Data
Source: PLoS One. 2010 Apr 1;5(4):e9993. doi: 10.1371/journal.pone.0009993 (PMC2848623; doi:10.1371/journal.pone.0009993)
Supplement: Text S1 — TiArA User Guide. This is a comprehensive manual detailing the installation and use of TiArA. (0.64 MB PDF) [file pone.0009993.s001.pdf]

TiArA (Ti)ling (Ar)ray Analyzer

September 17, 2009

Contents

1 Introduction **2**

2 Installation **2**
2.1 Virtual Machine Installation 2
2.2 Installation from Debian repository 3

3 Booting for the first time **3**
3.1 Configuring file sharing with the host (Virtualbox ONLY) 3

4 Using TiArA **4**
4.1 The TiArA interface 4
4.2 The analysis tab 5
4.2.1 Step 1 - Selecting a working directory 5
4.2.2 Step 2 - Selecting an array design 7
4.2.3 Step 3 - Selecting CEL files 8
4.2.4 Step 4 - Edit parameters 9
4.2.5 Step 5 - Edit analysis metadata 10
4.3 The export tab 11
4.3.1 Save R Session 11
4.3.2 Genome browser track 12
4.3.3 ORF expression level 12
4.4 Sessions 13

5 File formats **13**
5.1 Input files 13
5.1.1 TPMAP (chip layout) 13
5.1.2 CEL (Raw image data) 14
5.1.3 Genbank (Genomic annotations) 14
5.2 Output files 14
5.2.1 R script files 14
5.2.2 XLS (ORF summary) 14
5.2.3 .data (Normalized data) 15
5.2.4 WIG (Genome browser track) 15

1 Introduction

TiArA is a program for the analysis of Affymetrix tiling array data. Its aim is to be a user-friendly graphical interface for the normalization and summarizing of data.

2 Installation

There are two methods for installing TiArA on your computer. The preferred method is by 2.1 that will run on any modern computer using a virtualization solution such as Sun Virtualbox, or VMware. Alternatively, if you are running Ubuntu linux (version 8.04 or higher), you can 2.2 and install from there. The advantage of installing the virtual appliance is that the machine is specifically configured to run the TiArA program and will receive updates automatically.

2.1 Virtual Machine Installation

The virtual machine is distributed in Open Virtualization Format (OVF) and can therefore be installed on any machine running virtualization software that is OVF compatible. This includes Windows, Linux, and MacOS machines. The machine was developed on Sun Virtualbox (version 3.0) and is pre-configured to set up file sharing with the host machine using Sun's Virtualbox Tools. It is therefore recommended to install the system under Sun Virtualbox. More advanced users will be able to install the system on other virtualization platforms and configure file sharing through a networked server.

The following instructions indicate how to install the machine under Sun Virtualbox (version 3.0 or later):

1. Download and install Sun Virtualbox for your computer by following the instructions at http://www.virtualbox.org.

2. Download the TiArA virtual appliance file (tiara.tar.gz) from http://tiara.liai.org.

3. Unzip the the tiara.tar.gz.

4. Run Virtualbox and select "Import Appliance" from the "File" menu. If this option is not there, make sure you are running version 3.0 or later by clicking on "Help->About Virtualbox".

5. In the "Appliance Import Wizard" window that appears, click the "Choose" button. Navigate to the location where you unzipped the "tiara.tar.gz", select the "tiara.ovf" file and click "Open".

6. Clicking "Next" will bring you to the "Appliance Import Settings" screen. You should not need to change anything here. Click on the "Import>" button to bring up a popup window asking you to agree to the license. If you agree, click on the "Agree" button and the import process will begin.

7. After the import process completes, an entry called “TiArA-VM” should appear in the main window of Virtualbox. From here, you should follow the instructions on 3.

2.2 Installation from Debian repository

A Debian repository is maintained that will always store the latest version of the software. To install from this repository, please follow these steps:

1. Add the following line to your “/etc/apt/sources.list” file:
   deb http://tiara.liai.org/apt hardy contrib
2. Refresh your package database:
   sudo apt-get update
3. Install the package:
   sudo apt-get install tiara
4. From here, you should proceed to the section 4.

3 Booting for the first time

NOTE: This section only applies to those users using the virtual machine version of TiArA.

Upon first boot, you will be prompted to change several settings including adding a user account and password. Please follow the prompts and wait for the virtual machine to reboot before starting the TiArA software. Additionally, you will want to configure file sharing with the host machine, so that you can access the files that you wish to analyze and save the output to your host machine.

3.1 Configuring file sharing with the host (Virtualbox ONLY)

In order to configure sharing with the host machine, you should follow these steps:

1. With the virtual machine running in windowed mode (i.e. not full screen), select “Shared Folders” from the “Devices” menu. This will bring up a “Shared Folders” window.
2. Click on the folder with the “+” sign to add a new shared folder.
3. From the “Folder Path” drop down menu, select “Other” and navigate to the folder that you would like to access on your host machine (e.g. your “My Documents” folder).
4. In the “Folder Name” field, enter a short name for the folder with no spaces or special characters (e.g., “shared”,”host”, etc.).

5. Click on “OK” in this window and then again in the “Shared Folders” window to return to the virtual machine.

6. Click on the “Configure File Sharing with Host” icon on the virtual machine deskop. This will prompt you to enter the name of the shared folder.

7. In this window, type the name that you entered in step 4 and click “OK”.

8. Now, folder sharing should be set up. You will see an icon on your desktop with the name of your share. Clicking on it should allow you to access the files on your host machine.

4 Using TiArA

TiArA has been designed with usability in mind and presents the user with a stepwise interface. Upon opening the program, the user is asked whether to start a new session or load data from a previous session. A session consists of a set of CEL files, and the parameters by which they are analyzed.

4.1 The TiArA interface

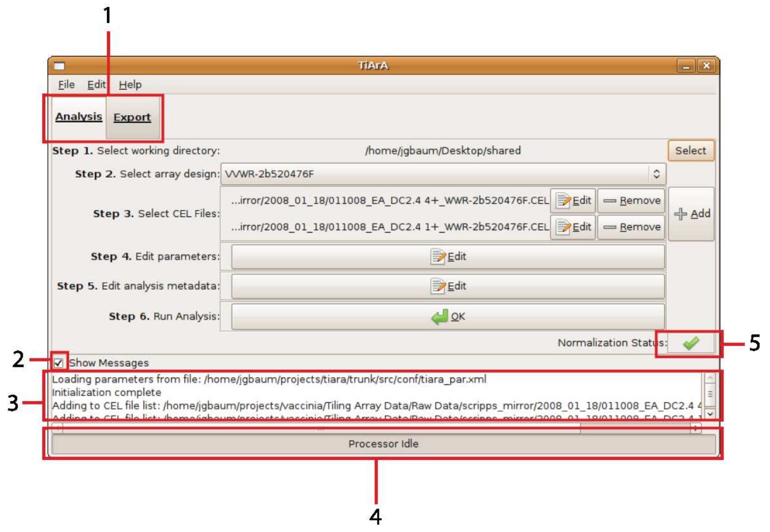

1. The tab selector - you can select either the “Analysis” tab or the “Export “ tab.

2. Show/Hide messages checkbox. Clicking on this box will toggle the display of verbose messages in the message box (3).

3. The message box - When visible, will display verbose messages regarding the analysis.

4. The progress indicator - This bar will display messages regarding the progress of the analysis.

5. Normalization status indicator - This indicates the status of the analysis. A red circle indicates that the data have not yet been normalized. A green checkmark indicates that the data are normalized and the export tab functionality is available.

Clicking on the “Start New Session” button will start TiArA with the default parameters. From there, the user should proceed through the steps on the “4.2”. As each step is completed, the next step becomes available. When you begin, only step 1 will be available. The remaining steps will be grayed out:

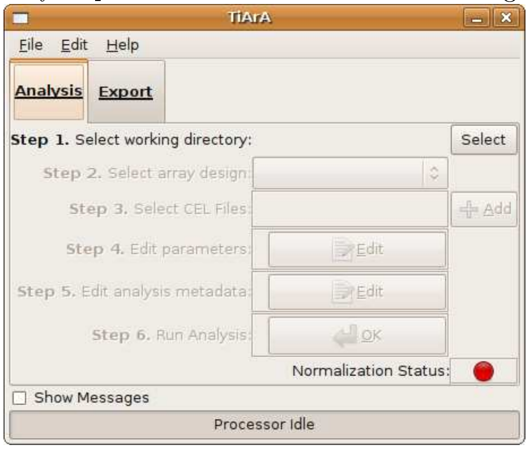

4.2 The analysis tab

4.2.1 Step 1 - Selecting a working directory

The first step is to select the working directory. This is where all of the files associated with your analysis will be stored. For each analysis, you should use a separate working directory to prevent new analyses from writing over old ones. To select a working directory, click on the “Select” button on the right of the window. This will bring up a file chooser dialog window where you can select the folder to use. If you would like to create a new folder, you may do so by clicking on “Create Folder”:

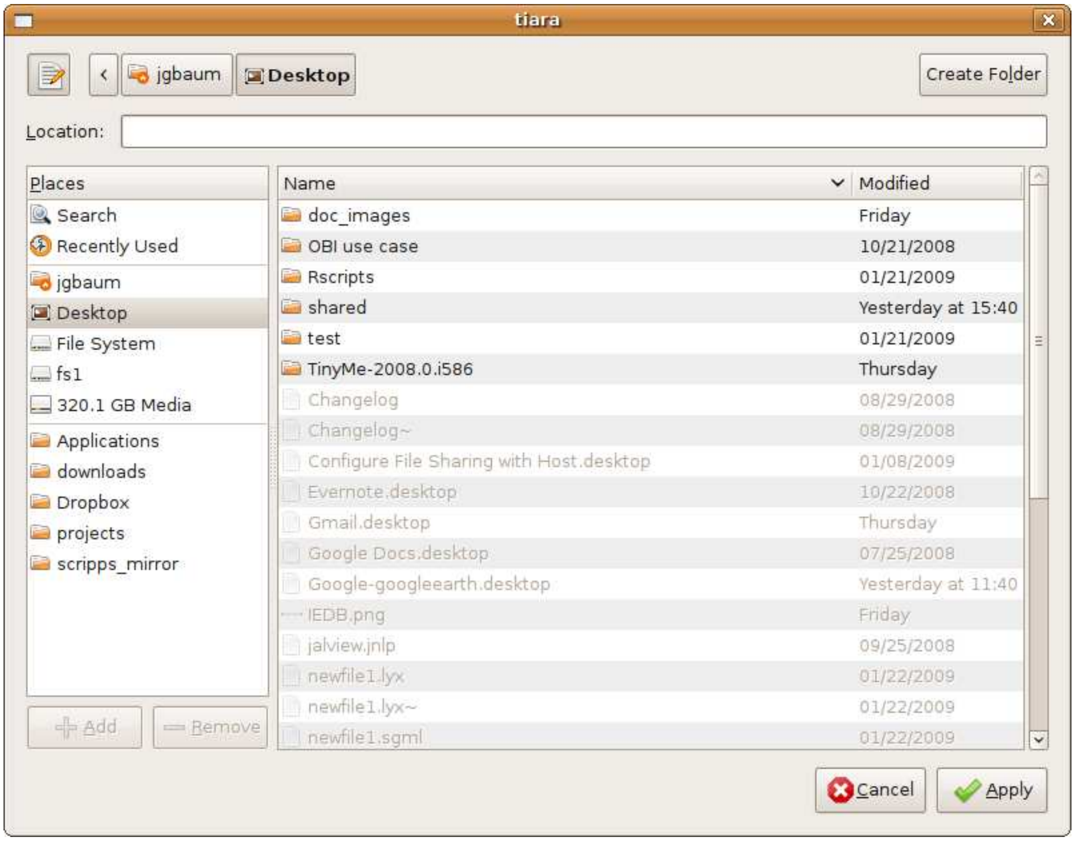

After you click "Apply", the working directory will be set and step 2 will become accessible:

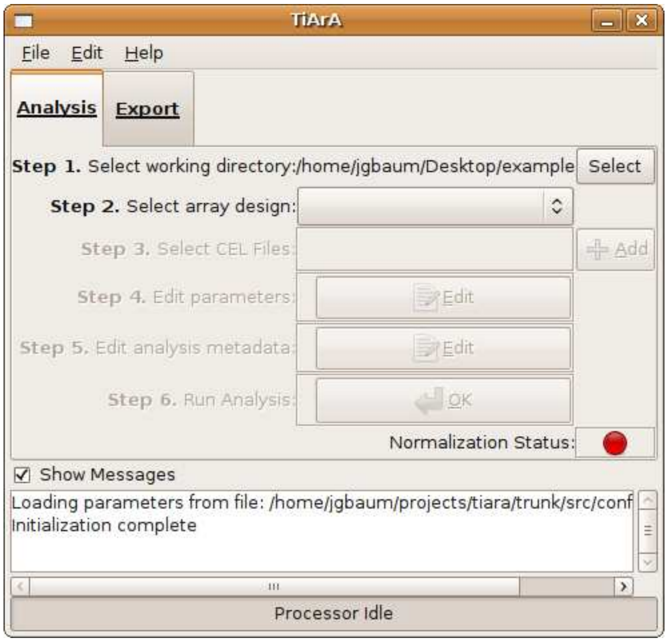

4.2.2 Step 2 - Selecting an array design

In order to analyze the data from your chip, the program needs to know the design of the array. The array design data is stored in a MySQL database back-end which is empty when TiArA is first installed. To tell the program about your array design, you must import a TPMAP file in this step. A TPMAP file is the text version of a BPMAP file and can be acquired by contacting Affymetrix directly. Once a TPMAP file is imported, the array design will be stored in the database and will be available for all future analyses. If you have already imported the TPMAP file you wish to work with, simply select it from the drop-down menu.

To import a TPMAP file, click on the drop-down box and select "New...". This will bring up the TPMAP import window:

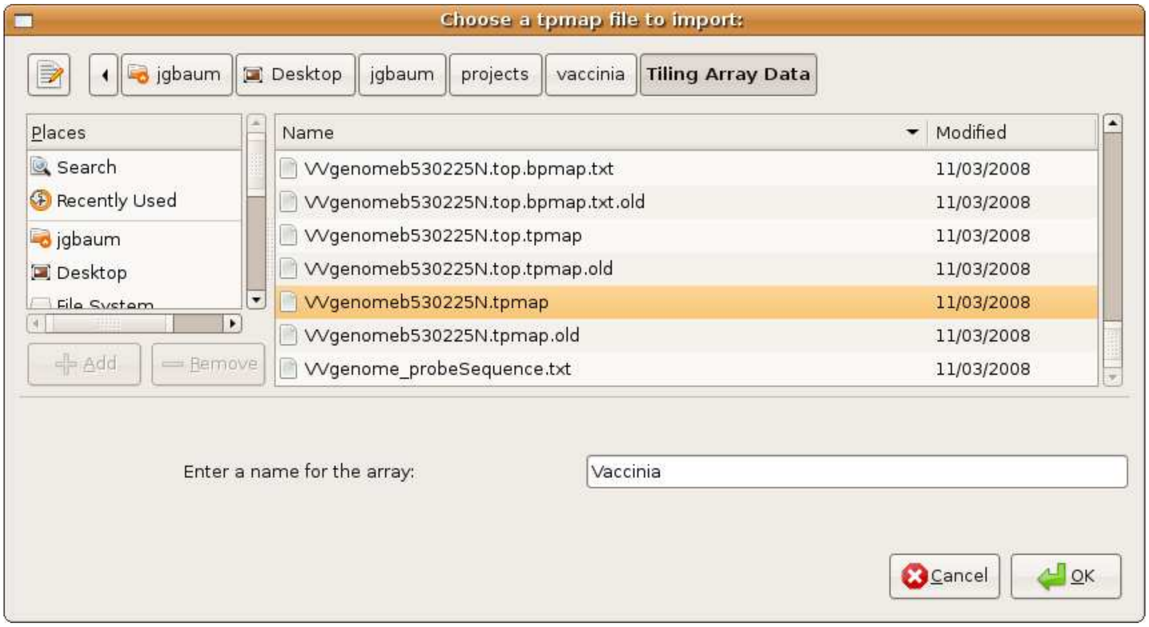

In this window, you should navigate to the TPMAP file that corresponds to your chip and enter a name for it in the text box. Clicking OK will cause the chip data to be read into the database. This can be a slow process, but only will be performed on this initial setup. If you would like to use the same array in the future, it will appear in the drop-down list under the name that you entered here.

4.2.3 Step 3 - Selecting CEL files

Probe intensity data for Affymetrix tiling arrays are stored in CEL files. To analyze a group of CEL files together, you must add them in this step. Clicking on the “Add” button on the right side of the window will bring up a file chooser. From here, you may select a set of CEL files to analyze. Note that if the CEL files are contained in different directories, you will have to add them from each directory separately. After you have selected the CEL files to analyze, the window should look similar to this:

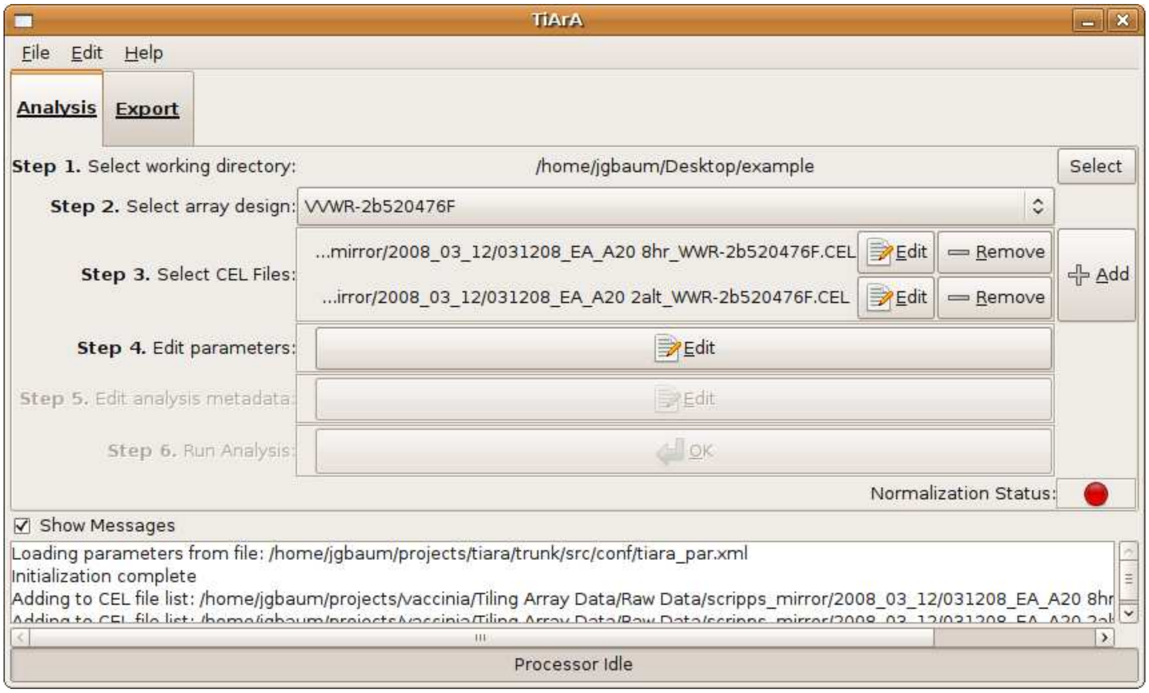

Next to each CEL file, two new buttons will appear, "Edit" and "Remove". The "Remove" button simply removes the CEL file from this analysis. The "Edit" button opens up a window that allows you to edit the metadata for the sample:

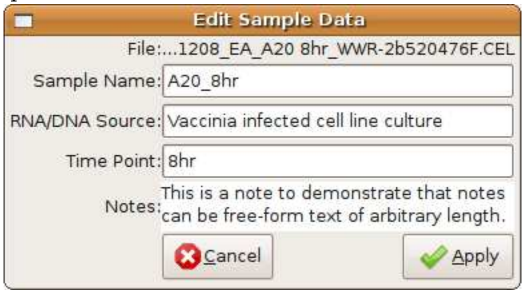

When the window first pops up, the fields will be empty except for the sample names, which are assigned default values. You do not have to edit the metadata, but it is encouraged to change the sample names to something meaningful so that the final output is understandable.

4.2.4 Step 4 - Edit parameters

In this step, you will choose the probe sets that will be analyzed. You must select at least one probe set to be analyzed and one probe set to use for background subtraction. Ideally, the probe set used for background subtraction will be non-specific and contain a sampling of different GC contents. Clicking on the "Edit"

button in step 4 will bring up the following window:

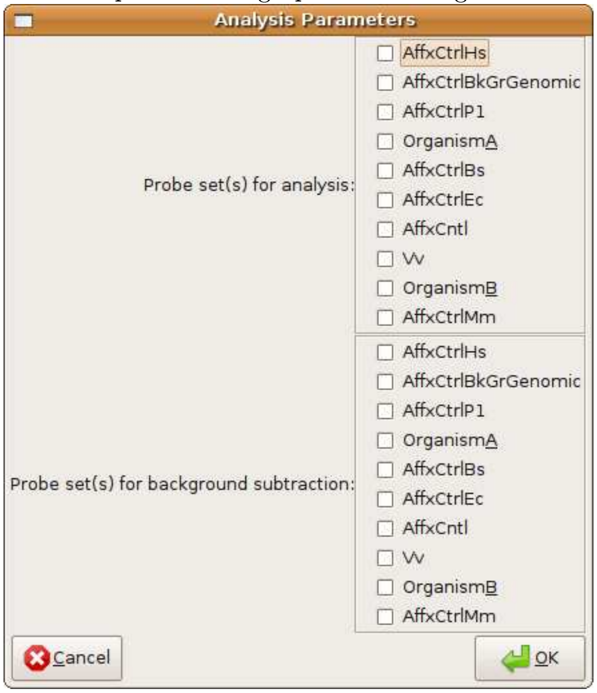

In this window, each of the probe sets from the TPMAP file will be listed. Once you have selected your probe set(s) for analysis and background subtraction, click "OK".

When this step is completed, TiArA will ask you if you would like to save your session. Please see 4.4 for further explanation.

4.2.5 Step 5 - Edit analysis metadata

This is an optional step to add certain metadata to your analysis. Currently, the only place this is stored is in the session file. Clicking on the "Edit" button in this step will bring up this window:

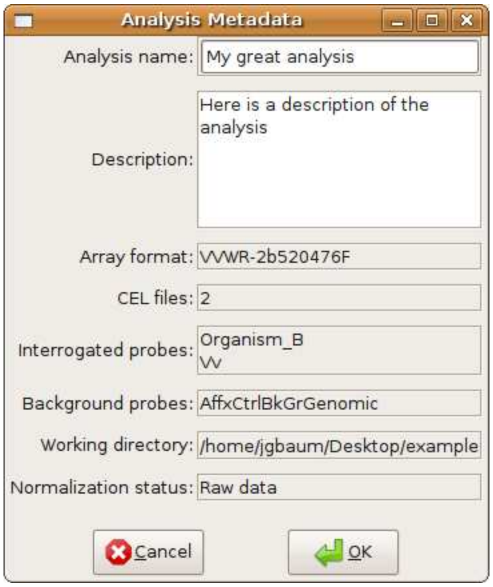

Step 6 - Analyze

After all of the other steps have been completed, clicking on the "OK" button in step 6 will perform the following:

1. Read CEL files into memory
2. Perform GC-based background subtraction
3. Write files (.data) for import into R
4. Import data into R
5. Quantile normalize array data against one another

This step will take some time depending on the number of arrays analyzed, size of the arrays, and processing power of your computer. Once it is complete, the normalization status indicator should change to a green check mark and the "Export" tab functionality will become available.

4.3 The export tab

This tab provides several means for exporting the data that were background-subtracted and normalized from the "Analysis" tab.

4.3.1 Save R Session

This button will save the current state of the R interpreter to a file, so that you may inspect the data manually and perform further analyses. To load this data into R, you would use the "load" function from within R.

4.3.2 Genome browser track

This will produce a file (WIG format) that can be viewed in a genome browser such as the Affymetrix Integrated Genome Browser (IGB). Clicking this button will bring up a window that prompts the user for several parameters:

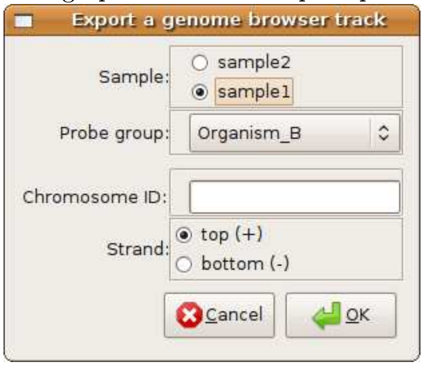

The user must select which sample to export, which probe group of the sample, a chromosome ID, and the strand. All parameters should be self-explanatory aside from the chromosome ID. The chromosome ID is the name of the chromosome as specified in the sequence selector of the IGB:

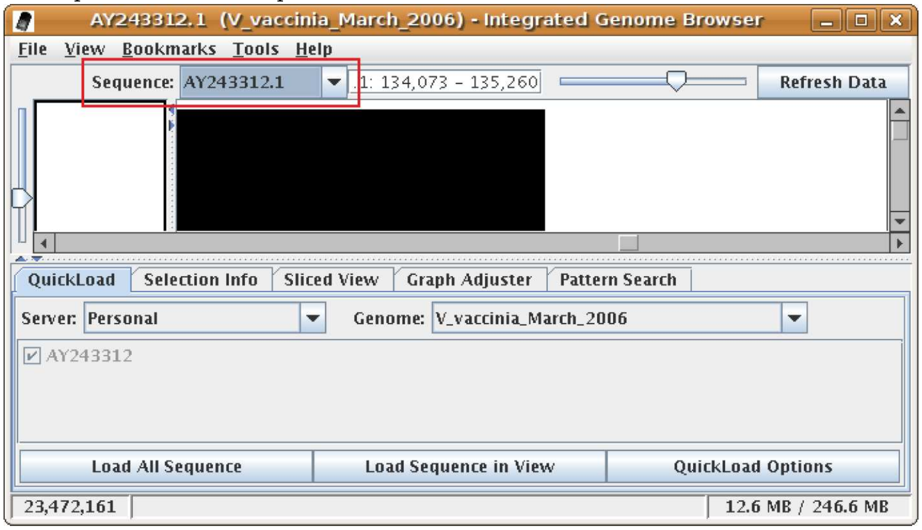

Once the file is exported, you can load it into IGB for viewing.

4.3.3 ORF expression level

Clicking on this button will allow you to calculate the expression level of each ORF/gene in the sequence. It can read in a Genbank file with annotations and will output a table of ORFs including their expression level and P-value. In order to use this feature, you must first set a few options:

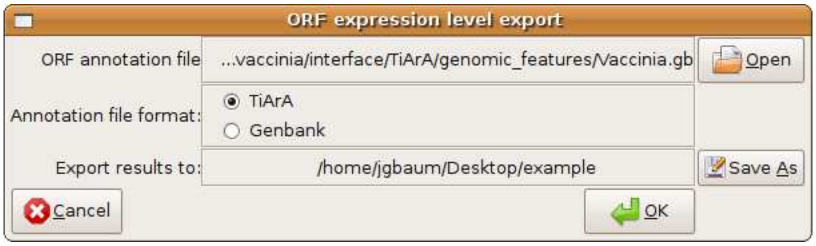

First, you must select an annotation file to use and the format of the file. TiArA can read files that are in Genbank format, as well as its own internal TiArA format. The results will be exported to the working directory by default, but this can be changed by clicking on “Save As”.

Once “OK” is pressed, the calculations will begin. This operation can take some time and is dependent upon the number and length of the ORFs which you are analyzing and the speed of your computer. Upon completion, a file titled “orf_expression.xls” will be exported to your working directory. This file will contain one worksheet per sample, named as you specified in the sample metadata (default=sample1, sample2, etc.).

4.4 Sessions

TiArA allows the user to save their progress in a file so that they can resume an analysis at a later time, or re-do an analysis with different parameters. The session file stores:

- analysis parameters
- CEL files
- analysis metadata
- sample metadata
- normalization state

The user will be asked at several points whether to save the session to a file. Additionally, the session can be saved at any point by clicking on “Save Session” from the “File” menu.

To load a session, the user can click on “Load a previous session” at startup or on “Load Session” from the “File” menu at any time.

5 File formats

5.1 Input files

5.1.1 TPMAP (chip layout)

The Affymetrix TPMAP file is a text version of the BPMAP file describing the layout of probes on the chip. If you do not have access to a TPMAP file, you can

use a utility included in the Affymetrix Power Tools to convert your BPMAP file to text.

5.1.2 CEL (Raw image data)

CEL files contain the raw image data from the chip.

5.1.3 Genbank (Genomic annotations)

Genbank files contain genomic sequence and annotation data including gene locations.

5.2 Output files

5.2.1 R script files

These files (ending in .R) will be found in the "Rscripts" directory underneath the working directory. These files are read into the R interpreter by the program, but can also be used for exploratory data analysis.

- There are 3 "process" files: process1.R, process2.R, and process3.R that include instructions for reading in the array format and .data files, as well as performing quantile normalization.
- If you summarize the ORF expression data, a file called "orf.R" is produced that includes instructions for creating this ORF summary.

5.2.2 XLS (ORF summary)

Calculating the level of ORF expression yields an Excel spreadsheet that can be viewed in Microsoft Excel, or OpenOffice. The file will have as many worksheets as there are samples in the analysis with the following fields:

- name - the name of the ORF/gene
- start/end - the starting and ending position of the ORF/gene in the context of the genome
- strand - the orientation of the gene. For historical reasons, "t" indicates the top, or forward, strand, while "f" indicates the bottom, or reverse, strand.
- seqid - the sequence identifier from the Genbank file
- N - the number of probes lying completely within the ORF/gene
- median - the median probe intensity within the ORF/gene
- mean - the mean probe intensity within the ORF/gene
- sd - the standard deviation of all of the probes within the ORF/gene

- P - the P value that the ORF/gene is expressed above background (calculated using the binomial distribution)

5.2.3 .data (Normalized data)

These files are created after the GC normalization and include 5 tab-delimited columns:

- uid - unique identifier for each probe
- pm_signal - the raw signal of the perfect-match probe
- pm_gcnorm - the GC normalized signal of the perfect match probe
- mm_signal - the raw signal of the mismatch probe
- mm_gcnorm - the GC normalized signal of the mismatch probe

Note that all signal intensities are log-transformed

5.2.4 WIG (Genome browser track)

This file includes the quantile normalized data for viewing in a genome browser. The format specification can be found at the UCSC website.
